# Supplementary material for: Improved Reference Genome Uncovers Novel Sex-Linked Regions in the Guppy (Poecilia reticulata)
Source: Genome Biol Evol. 2020 Aug 27;12(10):1789–805. doi: 10.1093/gbe/evaa187 (PMC7643365; doi:10.1093/gbe/evaa187)
Supplement: evaa187_Supplementary_Data [file evaa187_supplementary_data.zip › Supplemental Materials GBE.pdf]

Supplemental Materials:

Genome assembly:

Comparison of phases

We included sequences present in phase1, but not phase0, as additional contigs (termed decoys), in our mapping pipeline. To examine whether phase1 decoys were potentially biasing the downstream population genetic analyses, we examined coverage of reads to decoy contigs using the same coverage analysis as for the phase0 chromosomes, except that decoys were not trimmed, due to their small size. This analysis was performed with individuals from the GH population from which the genome was sequenced. Coverage of reads to phase1 decoy contigs was generally poor, with 18 and 23 decoys of 38 exhibiting normalised coverage below 0.8 in males and females, respectively, versus only 12 and 1 in phase0 in males and females, respectively. Five decoy contigs exhibited potential signs of sex-linkage, with male coverage greater 0.8 and improved on the phase1 decoy contigs compared with their corresponding phase0 regions. These regions were found at several genome locations, including LG10:1,842,142-1,845,153 bp (phase1 decoy M/F coverage ratio = 2.48, phase0 sex coverage ratio = 0.68), LG10:9,666,131-9,669,307 (phase1 decoy M/F coverage ratio = 3.07, phase0 M/F coverage ratio = 0.79), LG12:2,839,037-2,841,982 (phase1 decoy M/F coverage ratio = 2.12, phase 0 M/F coverage ratio = 0.80), LG12:23,456,655-23,459,300 (phase1 decoy M/F coverage ratio = 2.62, phase 0 M/F coverage ratio = 0.63), and LG8:15,930,097-15,933,408 phase1 decoy M/F coverage ratio = 2.57, phase 0 M/F coverage ratio = 0.62). However, when this analysis was repeated with Oropouche individuals, no windows, including those on LG12, showed consistent male bias across both populations.

Table S1: Description of the raw and release genome assembly.

	**raw assembly**	**release assembly**
Number of contigs	401	415
Total size	739303373	745001348
N50 contigs	7935619	7936040
N90 contigs	1441918	1331878
Length of longest contig	25604303	25604303
Length of shortest contig	20028	20028

Table S2: A comparison of mapping rates across different versions of the guppy genome; the previously published female genome (FG), phase 0 of the raw assembly, phase 1 of the raw assembly, and the release assembly. For each population sampled, we summarised the mean per sex of percent of paired-reads mapped properly.

Population	FG genome	MG Phase 0 raw assembly	MG Phase 1 raw assembly	MG release assembly
HPG females	93.02	97.08	97.06	97.53
HPG males	90.14	95.95	95.59	96.36
LPG females	92.84	97.00	96.97	97.45
LPG males	90.79	95.28	95.26	95.68
HPO females	91.49	95.08	95.07	95.09
HPO males	91.01	94.90	94.89	94.70
LPO females	91.50	94.95	94.93	94.83
LPO males	91.35	94.92	94.91	94.83
HPM females	91.33	94.70	94.68	94.68
HPM males	91.45	94.88	94.86	94.55
LPM females	91.10	94.44	94.41	94.05
LPM males	90.95	94.52	94.50	94.60

Table S3: Repeat annotation for the male genome (MG) as identified by RepeatMasker ver (open-4.0.5).

	Number of elements	length occupied (bp)	percentage of sequence (%)
Retroelements	71565	27444545	3.68
SINEs:	21546	4586421	0.61
Penelope	265	63739	0.01
LINEs:	39941	16071894	2.15
CRE/SLACS	0	0	0
L2/CR1/Rex	19853	4926421	0.66
R1/LOA/Jockey	2612	910621	0.12
R2/R4/NeSL	2960	2345010	0.31
RTE/Bov-B	11803	6195573	0.83
L1/CIN4	2189	1477364	0.2
LTR elements:	10078	6786230	0.91
BEL/Pao	958	1367916	0.18
Ty1/Copia	95	181731	0.02
Gypsy/DIRS1	5391	4500220	0.6
Retroviral	738	240562	0.03
DNA transposons	161863	26827179	3.59
hobo-Activator	34983	3942439	0.53
Tc1-IS630-Pogo	104251	20565875	2.76
En-Spm	0	0	0
MuDR-IS905	0	0	0
PiggyBac	423	84459	0.01
Tourist/Harbinger	1368	157179	0.02
Other (Mirage, P-element, Transib)	1	73	0
Rolling-circles	0	0	0
Unclassified:	8754	1427650	0.19
Total interspersed repeats:		55699374	7.46
Small RNA:	3622	768365	0.1
Satellites:	3520	815424	0.11
Simple repeats:	289506	14785307	1.98
Low complexity:	41722	2048748	0.27

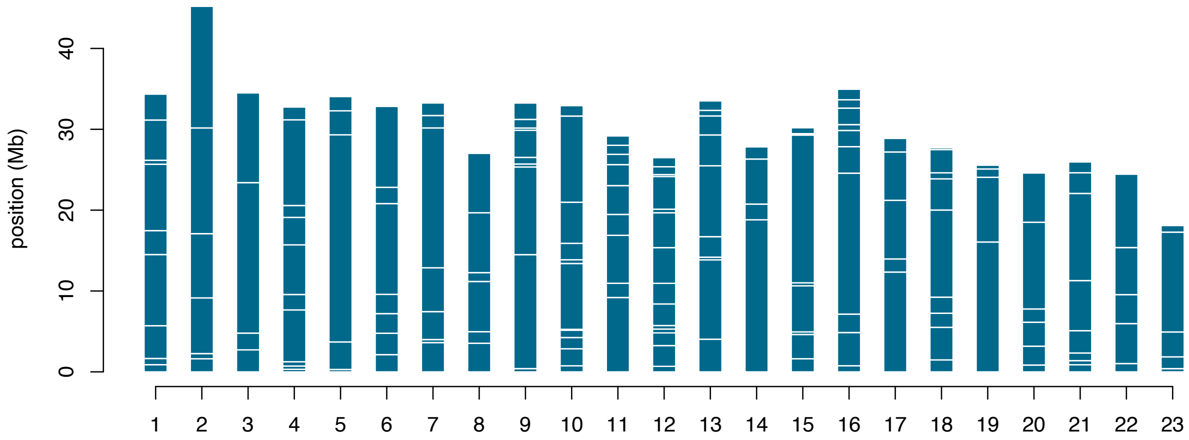

Figure S1: Distribution of contigs placed across chromosomes. Contigs are represented as blue boxes.

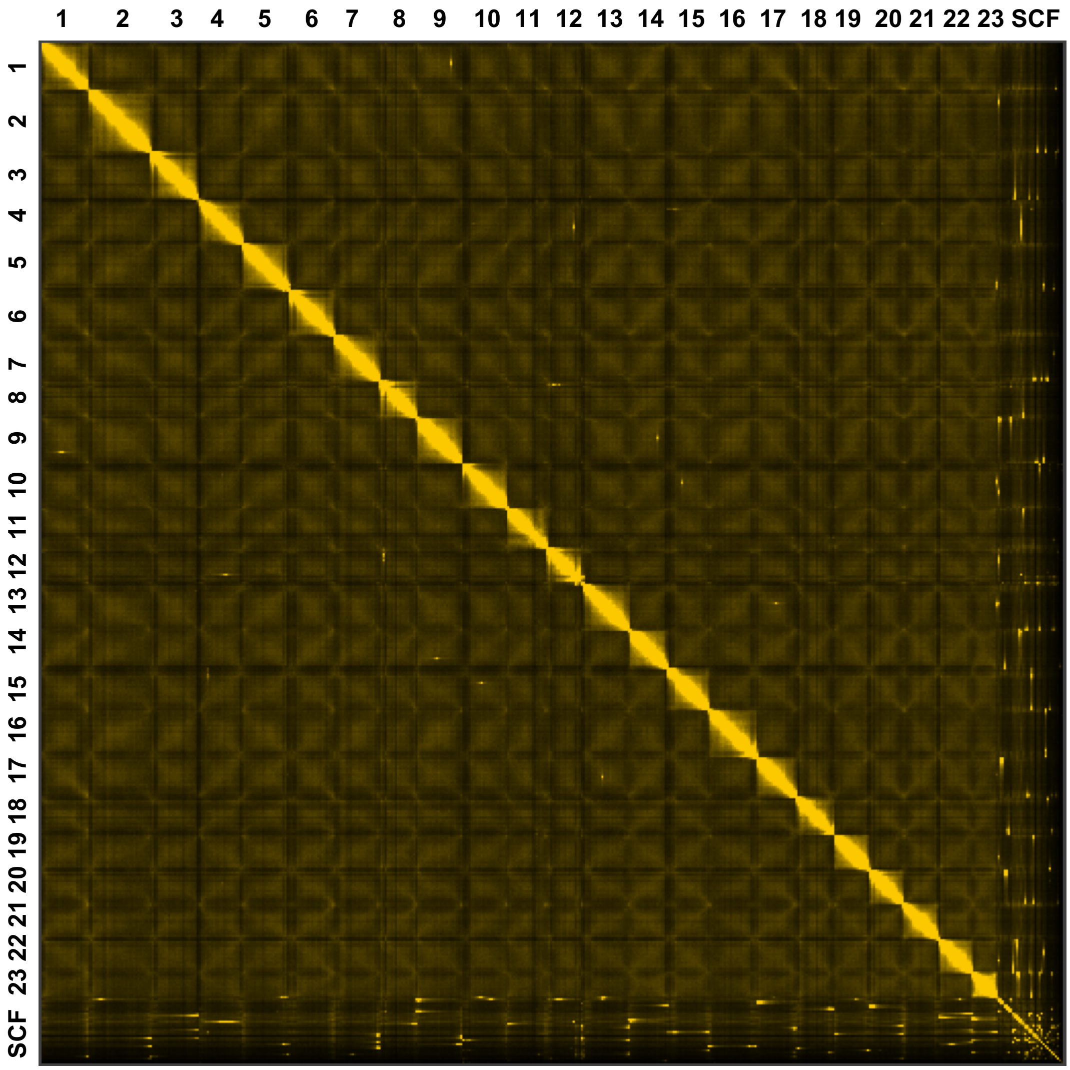

Figure S2: Whole genome Hi-C chromosomal contact information, each chromosome is numbered while unplaced scaffolds are placed together.

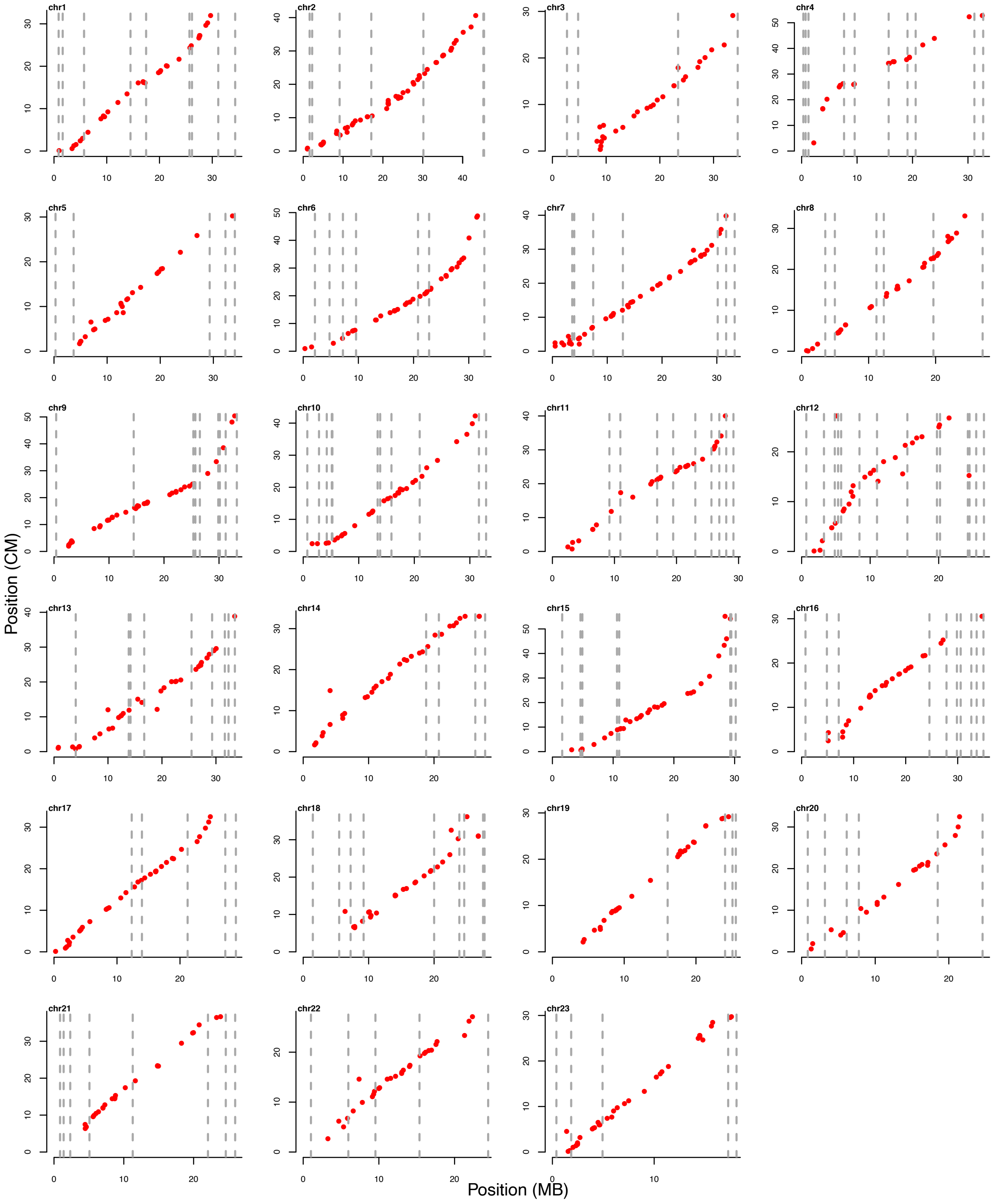

Figure S3: Genetic linkage map 1 (GM1), with each marker's position shown on the MG genome in centimorgans (cM). Dashed lines indicate contig boundaries.

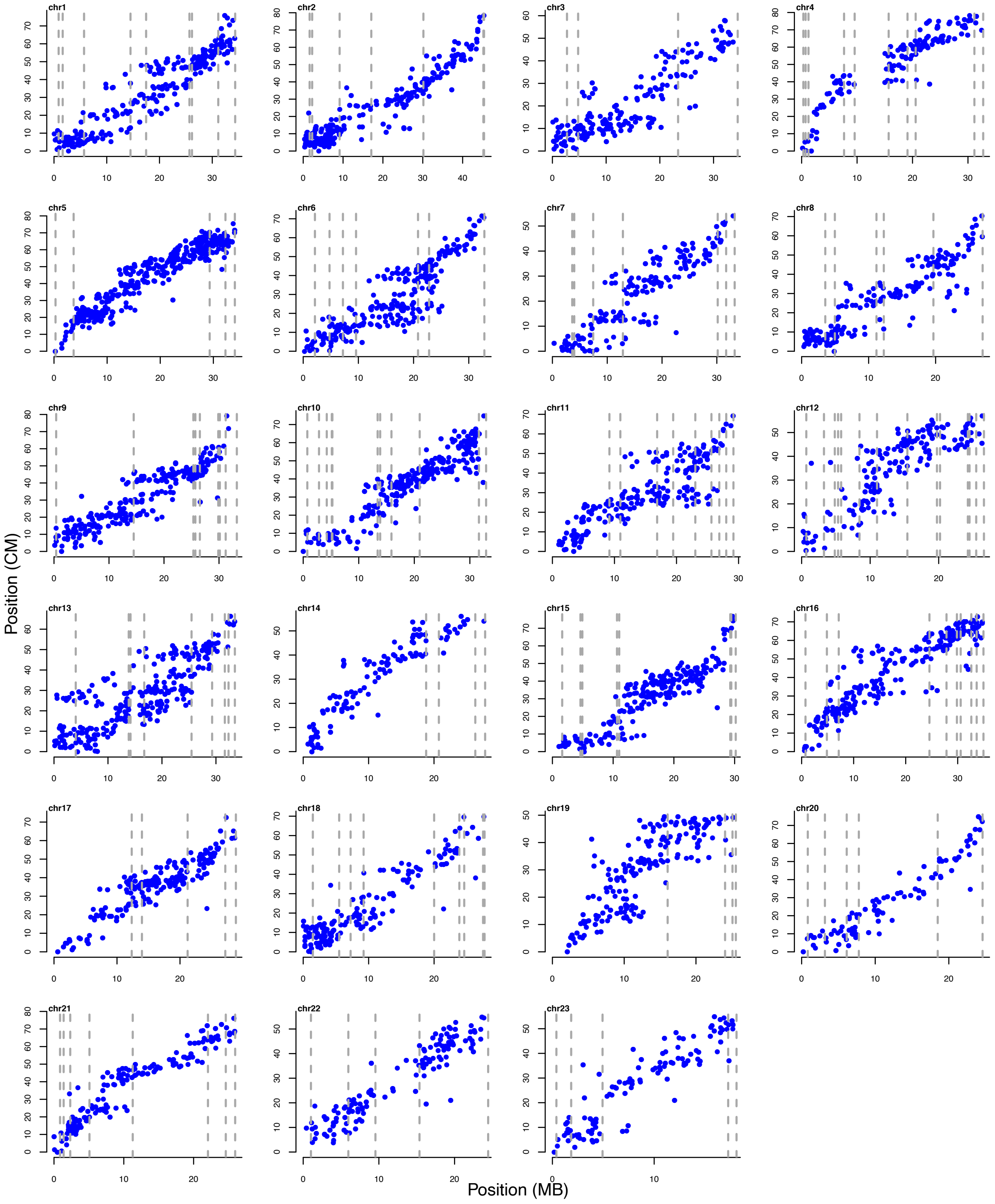

Figure S4: Genetic linkage map 2 (GM2), with each marker's position shown on the MG genome in centimorgans (cM). Dashed lines indicate contig boundaries.

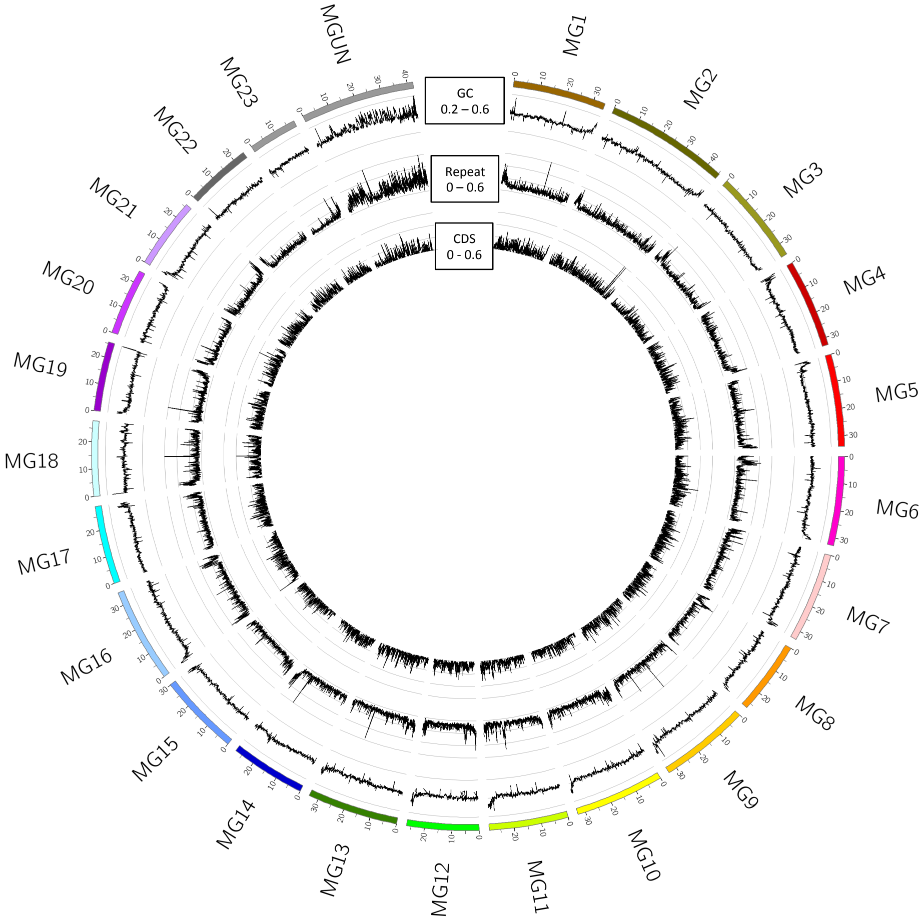

Figure S5: Whole-genome distributions of GC content (outermost track), repeat content (middle track), and genic content (innermost track). Each chromosome is shown as well as unplaced contigs as chrUN.

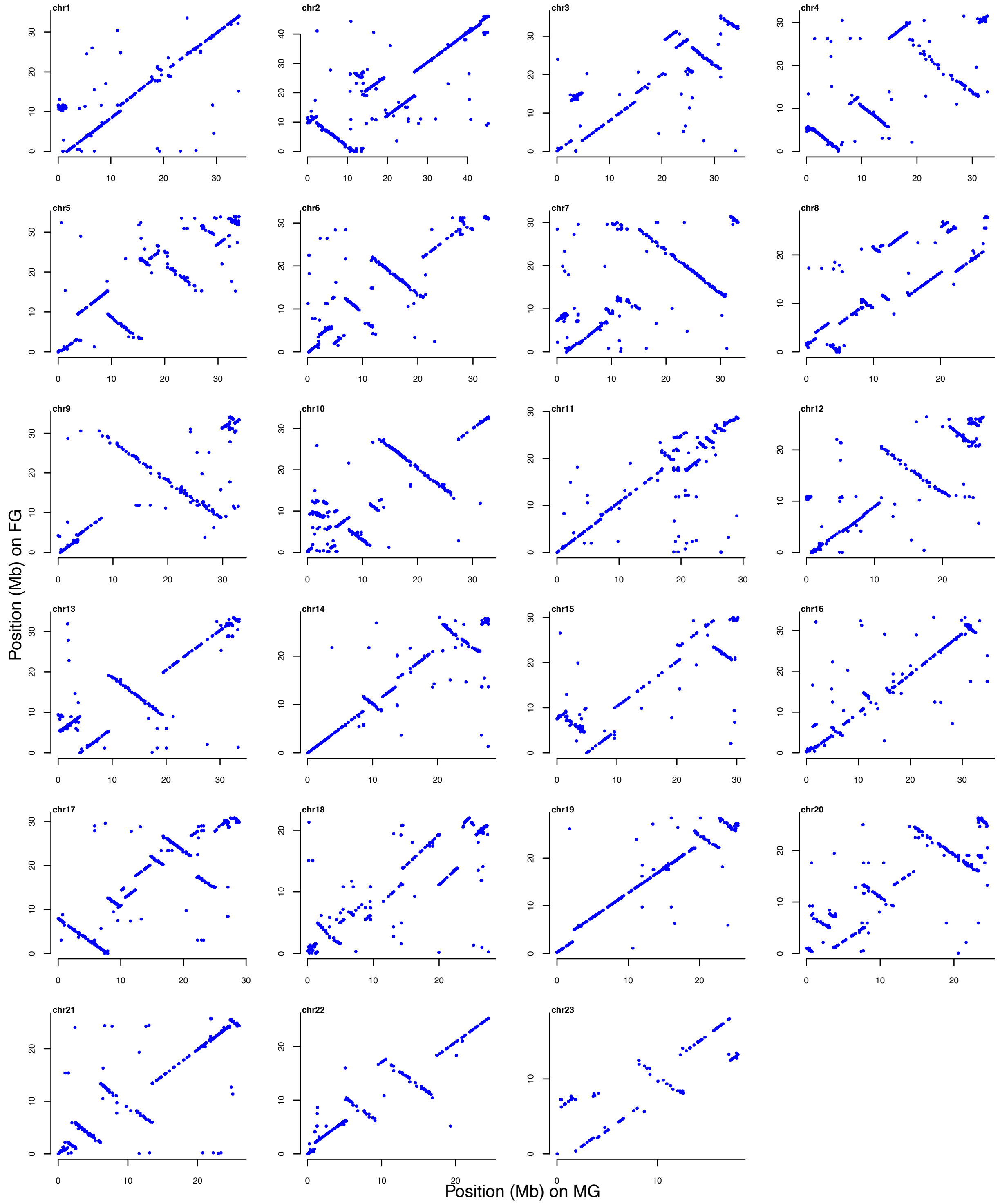

Figure S6: Whole-genome alignments between the male genome (MG) and previously published female genome (FG) assemblies for each chromosome. Only alignments in regions with high quality are shown (i.e. mapping quality ≥ 60).

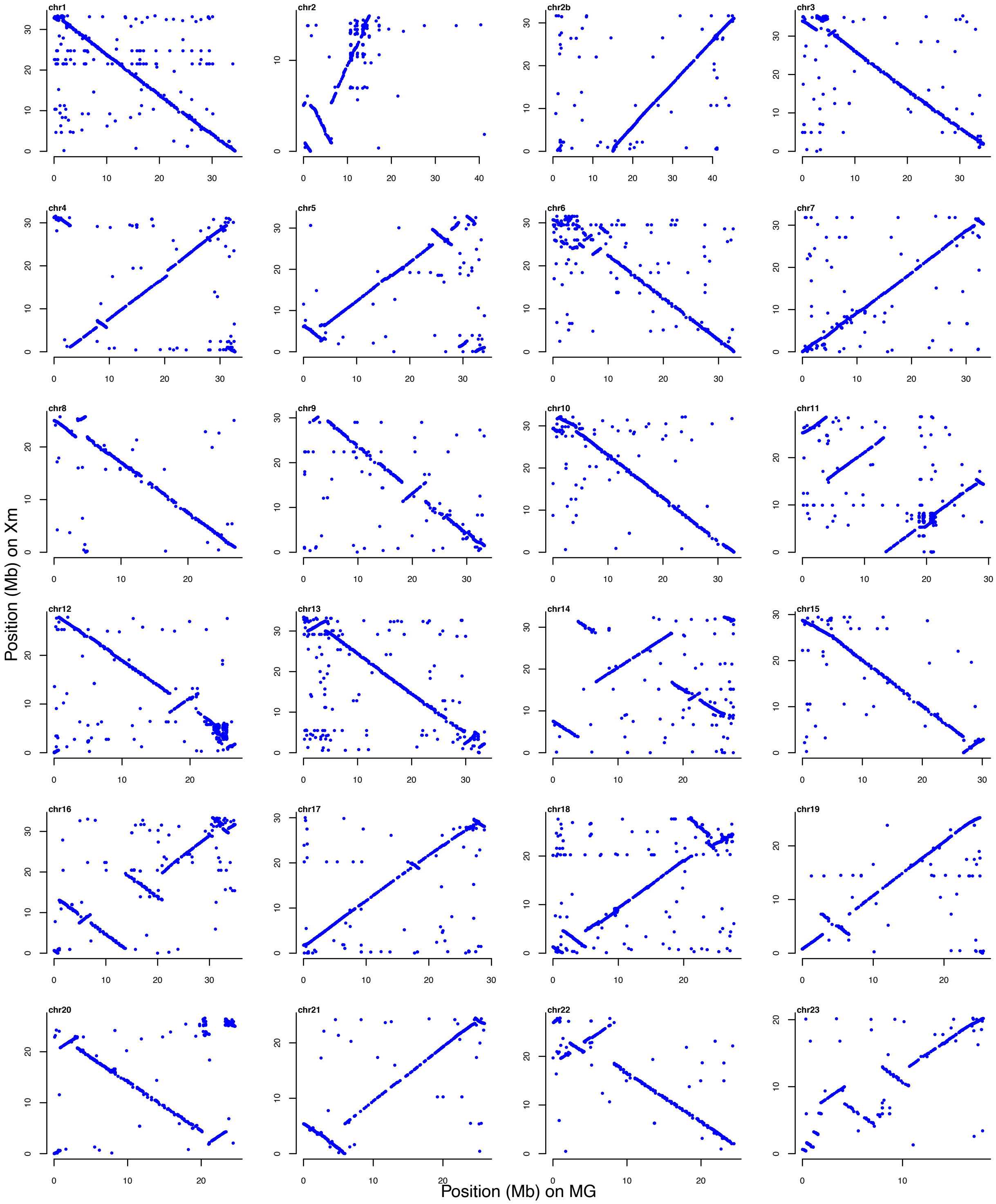

Figure S7: Whole-genome alignments between the updated male genome (MG) and *Xiphophorus maculatus* (Xm) assemblies for each chromosome. Only alignments in regions with high quality are shown (i.e. mapping quality ≥ 60).

Examination of LG12:
After placing contigs along linkage groups, we revisited the HiC in more detail (figure S8). We rearranged a few contigs so that the ordering was in line with the HiC data. Specifically, we inverted the first two contigs, moved contig V to be adjacent to contig IV, inverted contig XI, and reordered contig XIII and XIV.

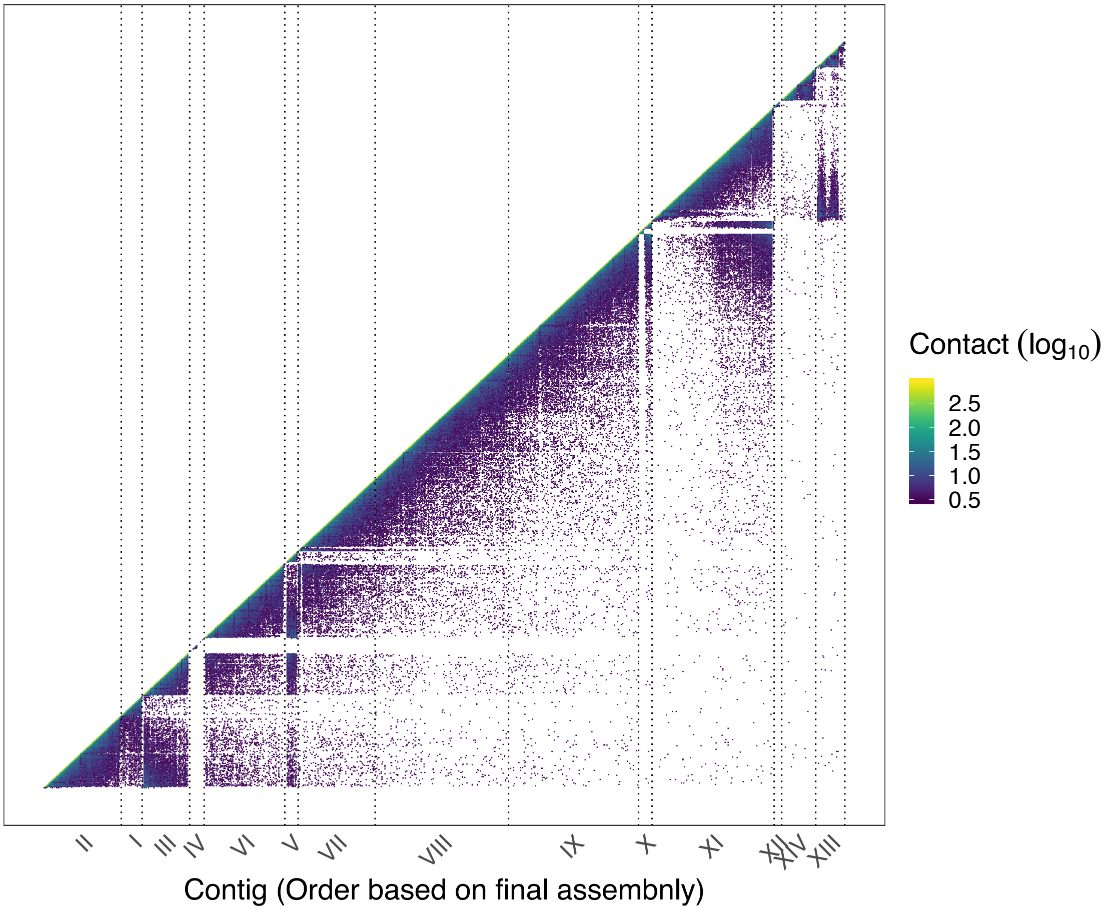

Figure S8: Assembly and HiC contact information of LG12 before manual curation, with each contig being delineated with dashed lines and numbered as in main text, i.e. final arrangement.

Table S4: Comparison of phase 0 and phase 1 contigs on LG12. Shown are the contig names, corresponding to figure 2, start and end position in the release genome assembly, genetic distance between the phases, and size differences between the phases (phase 0 - phase 1). NA refers to contigs that were changed between the raw and release assembly and therefore do not have corresponding phases.

Contig ID	Start position	End position	Genetic Distance (SNPs/Contig Length)	Size difference between phases (bp)
I	1	682334	1.00E-02	13667
II	692334	3258872	1.22E-02	-25197
III	3268872	4838750	1.11E-02	31525
IV	4848750	5321818	3.22E-03	-117381
V	5331818	5765682	6.94E-03	4464
VI	5775682	8441695	9.32E-03	-62560
VII	8451695	11000781	9.62E-03	6090
VIII	11010781	15428900	9.37E-03	-11830
IX	15438900	19747371	9.56E-03	17486
X	19757371	20199082	*NA*	*NA*
XI	20209082	24252561	9.79E-03	8334
XII	24262561	24501363	1.22E-03	9
XIII	24511363	25475239	9.15E-03	7022
XIV	25485239	26605241	*NA*	*NA*

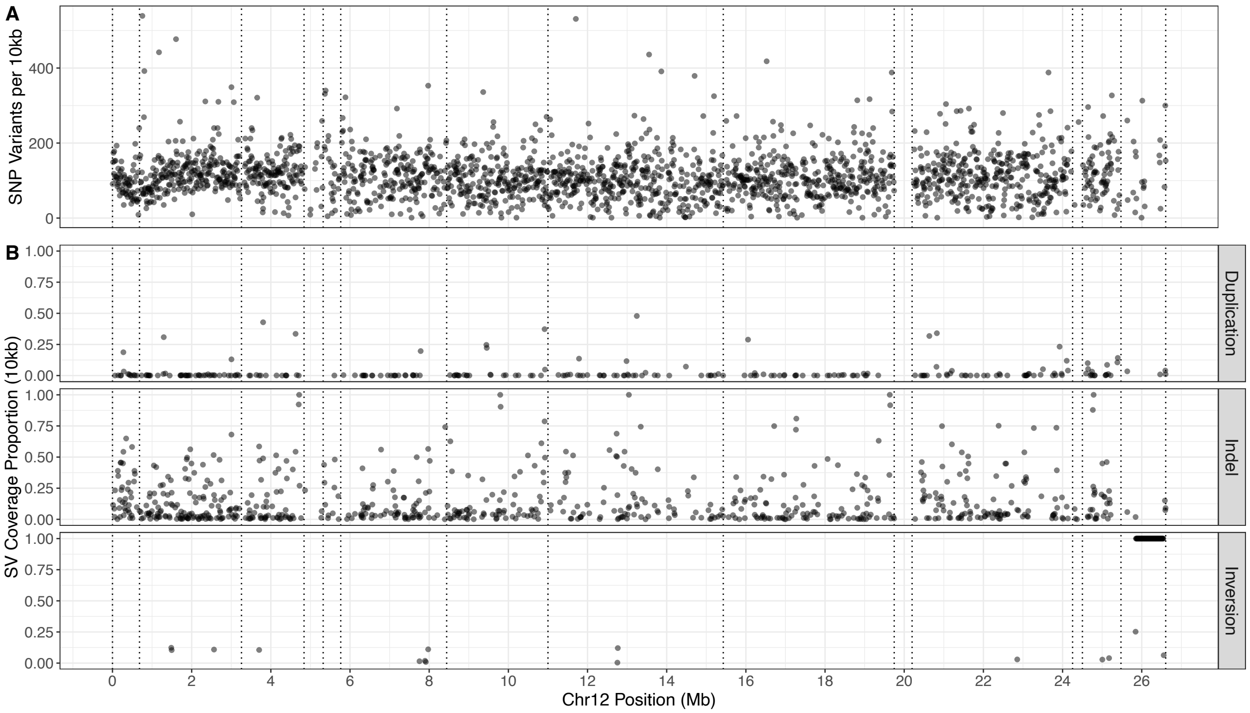

Figure S9: Differences between phase 0 and phase 1 across LG12. (A) SNP differences per 10kb, (B) Structural variant (SV) differences summarised per proportion covered by 10kb. Shown are duplications, indels, and inversions. Dashed lines indicate contig boundaries.

Population genomics

Table S5: Table summarising male and female samples from six wild populations and their mapping statistics to the MG genome. HP denotes high predation, LP denotes low predation populations. IDs of populations given in brackets.

Population	# of females	Female Samples<br>Mean # reads<br>Mean # mapped paired reads<br>Coverage<br>% paired reads mapped	# of males	Male Samples<br>Mean # reads<br>Mean # mapped paired reads<br>Coverage<br>% paired reads mapped
HP Guanapo (GH)	10	77,713,416<br>71,539,143<br>9X<br>97.53%	9	92,662,458<br>86,465,594<br>11X<br>96.36%
LP Guanapo (GL)	8	91,316,973<br>83,440,476<br>11X<br>97.45%	10	105,314,493<br>97,036,576<br>13X<br>95.68%
HP Oropuche (OH)	10	77,882,229<br>70,619,279<br>11X<br>95.09%	9	106,550,932<br>99,134,444<br>17X<br>94.70%
LP Oropuche (OL)	12	86,037,153<br>79,026,947<br>13X<br>94.83%	8	85,358,224<br>78,098,644<br>13X<br>94.83%
HP Marrianne (MH)	10	104,765,775<br>95,978,602<br>16X<br>94.68%	7	100,395,645<br>93,492,333<br>16X<br>94.55%
LP Marrianne (ML)	11	79,800,263<br>74,532,743<br>14X<br>94.05%	6	105,340,624<br>95,837,332<br>15X<br>94.60%

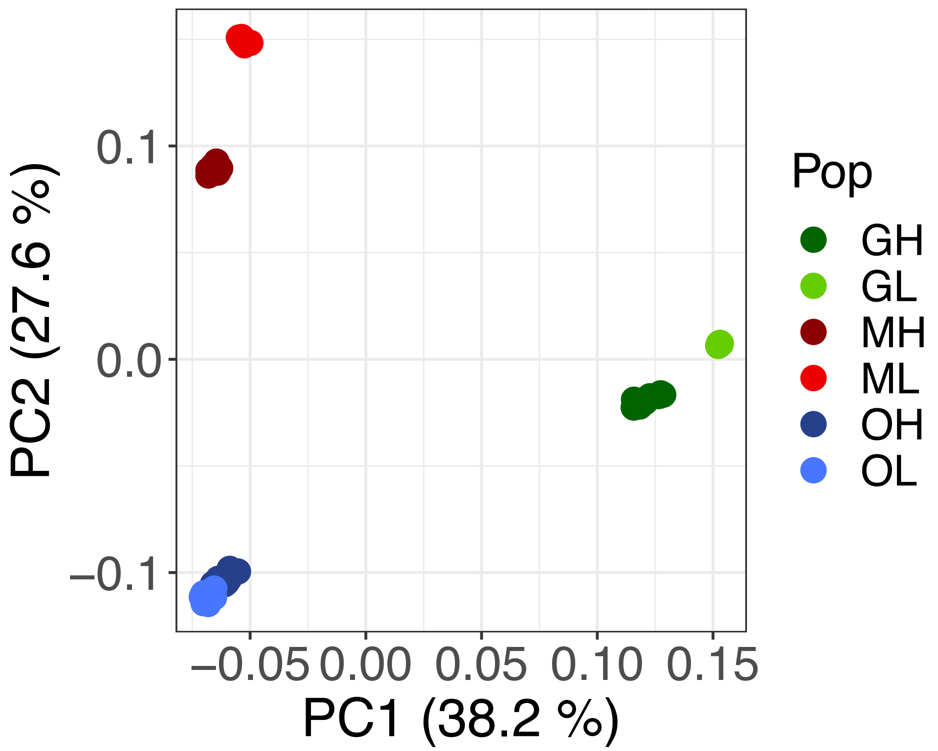

Figure S10: Principal component analysis of population samples using whole-genome diversity. Plotted are PC1 (with an eigenvalue of 38.2%) against PC2 (with an eigenvalue of 27.6%), each population is colour coded (see legend).

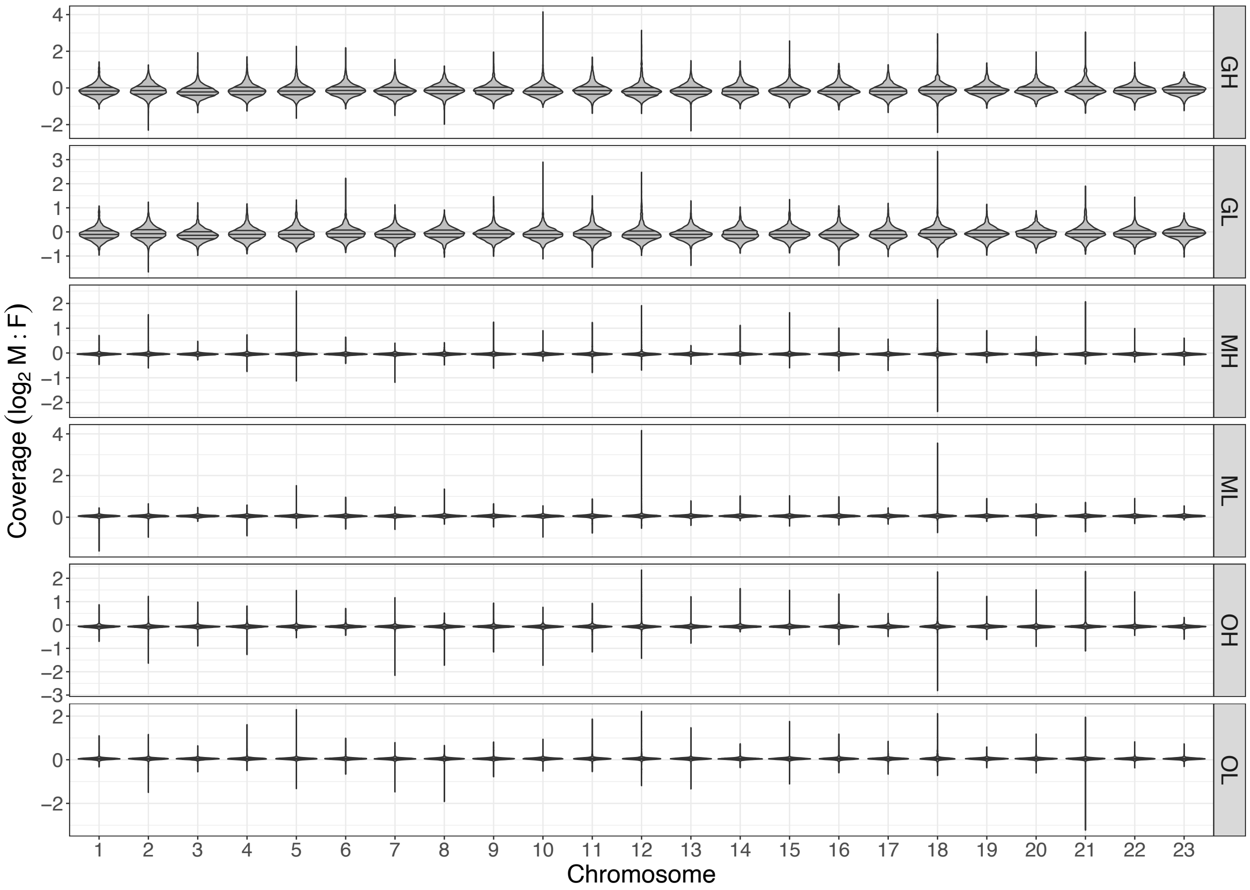

Figure S11: Coverage ratio between the sexes (log2[M:F]) across the genome. Shown are violin plots for each chromosome for each population sampled. Population abbreviations can be found in Table S5.

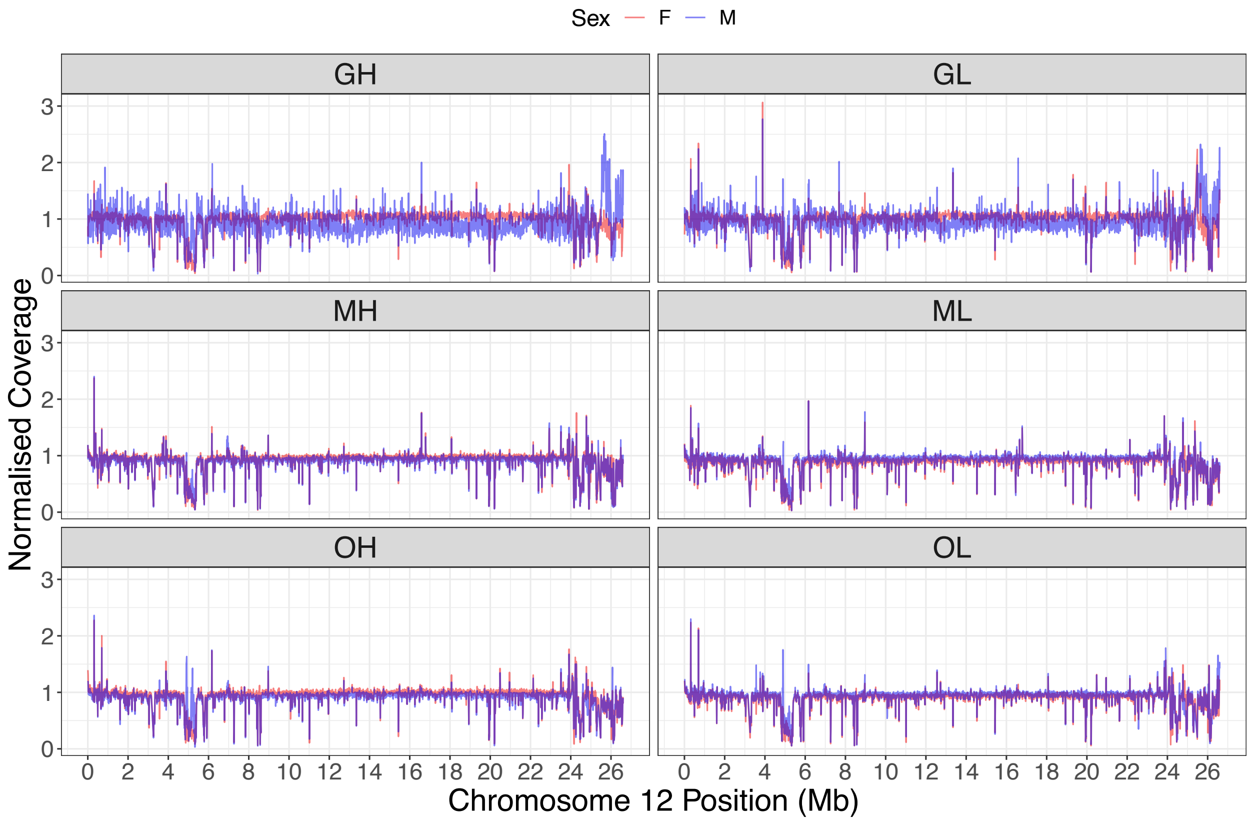

Figure S12: Normalised coverage for males and females separately across LG12. Each population is shown in a panel, with high-predation (HP) populations on the left and low-predation (LP) populations on the right. Coverage is fitted with a spline, males in blue and females in pink.

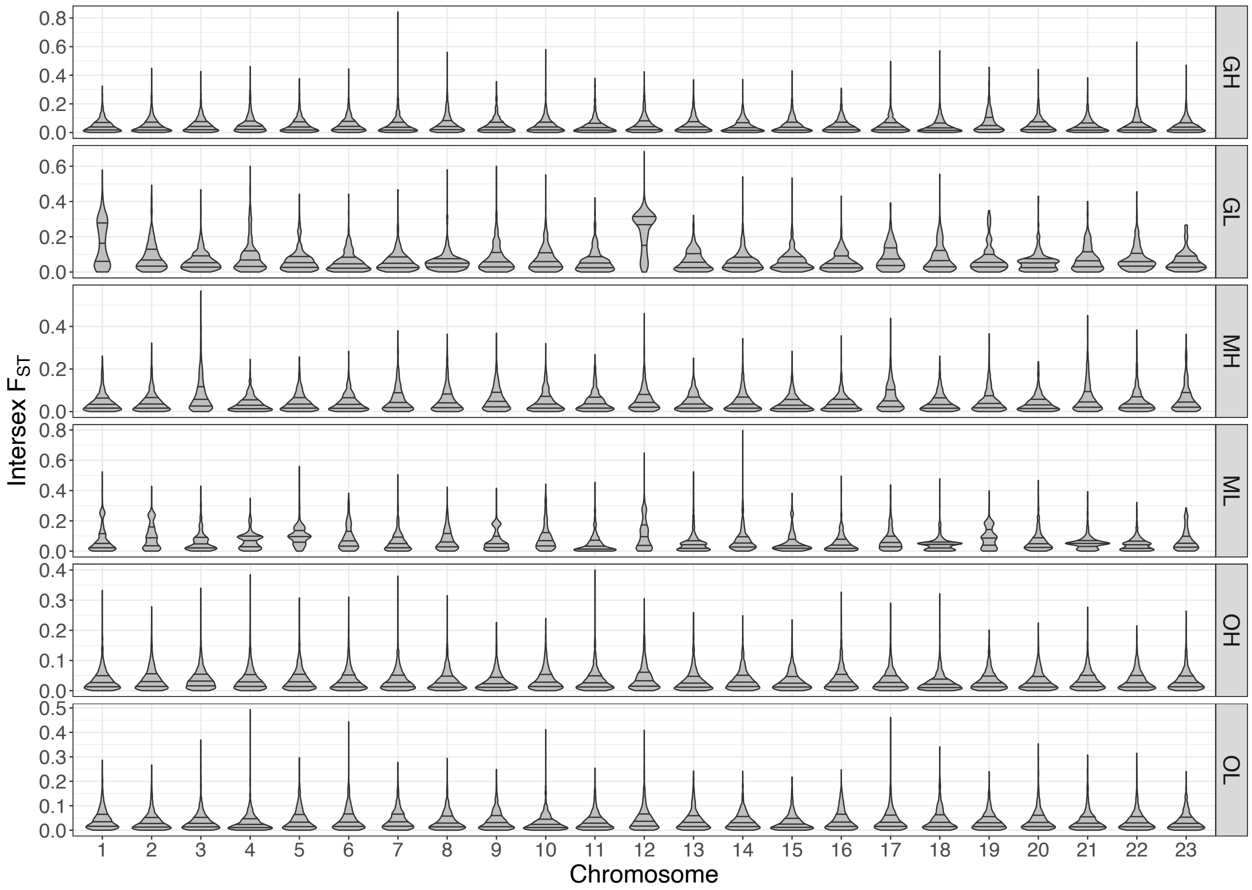

Figure S13: F_{ST} between the sexes across the genome. Shown are violin plots for each chromosome for each population sampled. Population abbreviations can be found in Table S5.

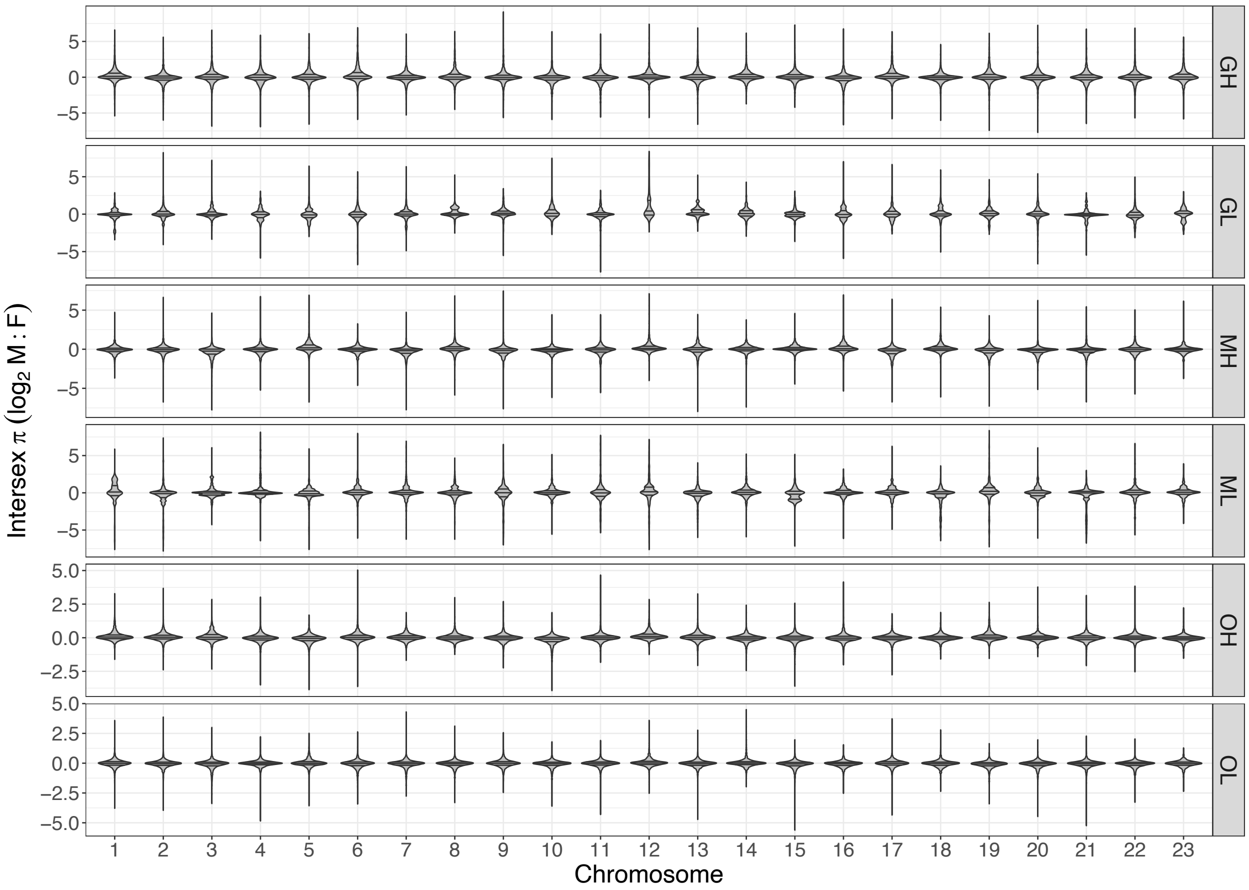

Figure S14: Nucleotide diversity (pi) difference between the sexes (log2[M:F]) Shown are violin plots for each chromosome for each population sampled. Population abbreviations can be found in Table S5.

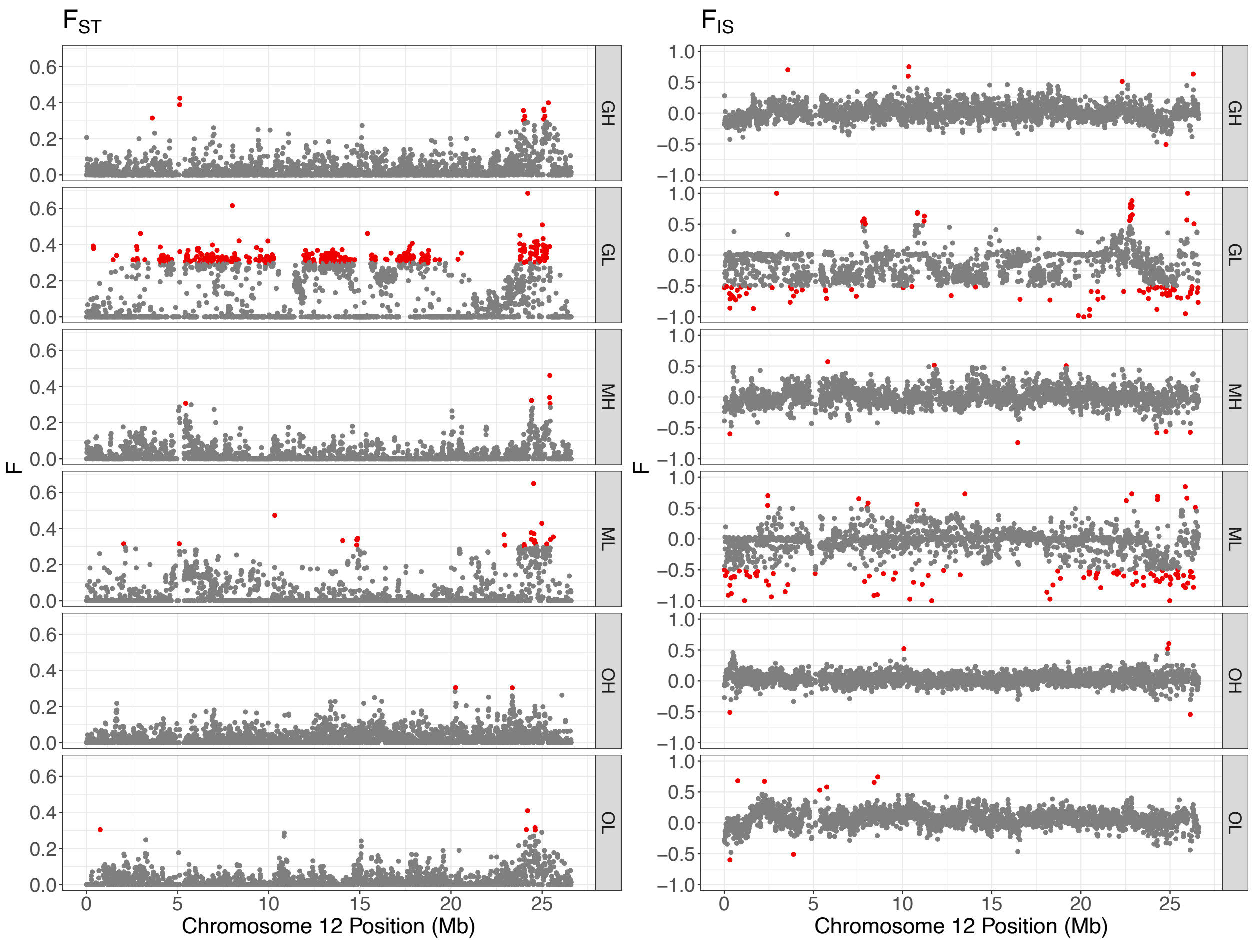

Figure S15: F_{ST} between the sexes along LG12 for each population studied, red points denote outliers of above 0.3. F_{IS} between the sexes along LG12 for each population studied, red points denote outliers of above or below 0.5.

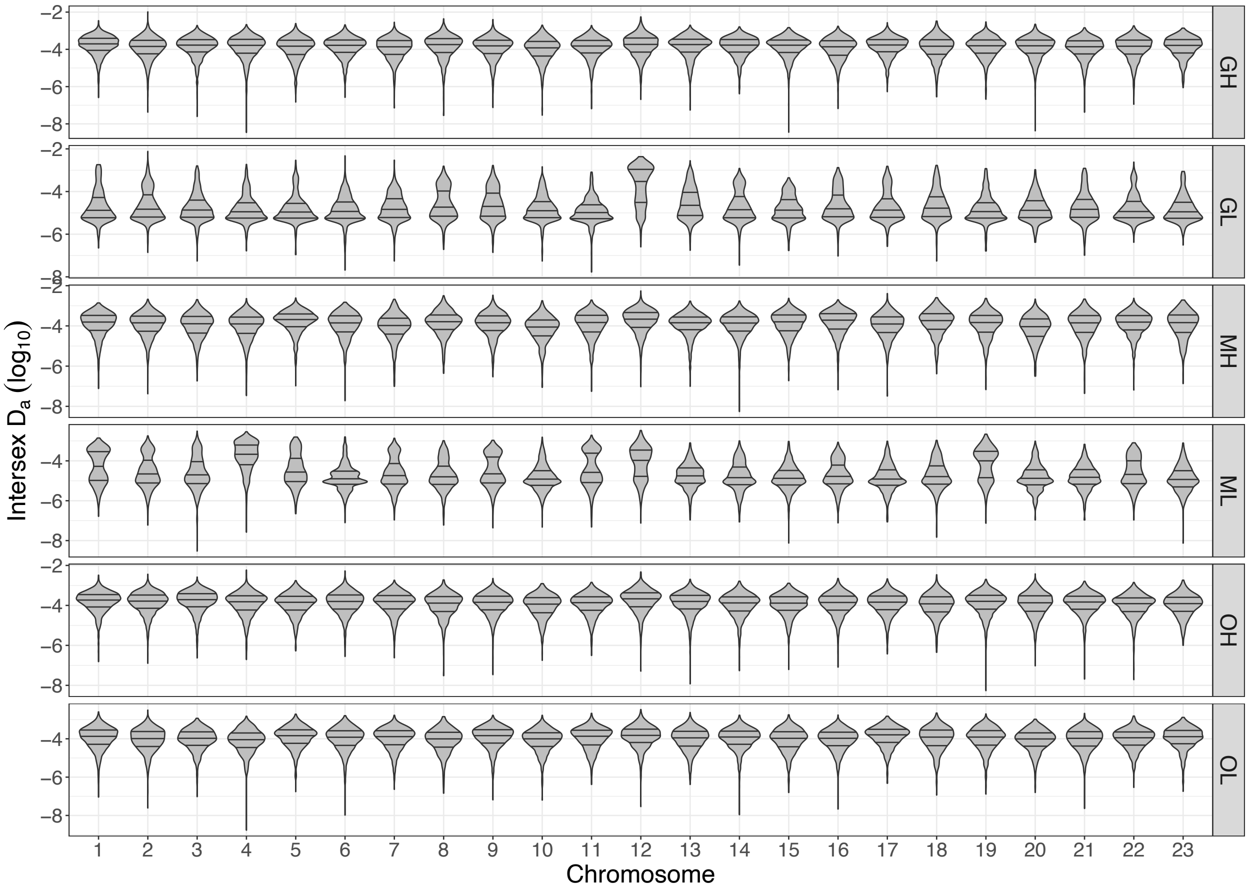

Figure S16: Net divergence (D_a) along the genome. Shown are violin plots for each chromosome for each population sampled. Population abbreviations can be found in Table S5.

Table S6: Overlapping outlier windows of coverage ratio between the sexes. Shown is mean normalised coverage for each sex for each population and the number of populations where that window was identified as an outlier (N_shared). (xlsx file)

Table S7: Overlapping outlier windows of F_{ST} between the sexes. Shown are F_{ST} values per population and the number of populations where that window was identified as an outlier (N_shared). (xlsx file)

Table S8: Overlapping Net diversity (D_a) outliers windows. Shown are D_a values per population and the number of populations where that window was identified as an outlier (N_shared). (xlsx file)

Y-mer results

After cleaning and error-correction, the average number of reads ranged from 85,938,590 (OL) to 318,421,470 (OH) in males, and the average number of reads in females ranged from 148,775,839 (OH) and 201,770,261 (MH). Between groups, males had a higher number of reads, except in the case of OL. Using the female filtered matrix, the mean number of k-mers ranged from 3 (OL) to 10 (OH) and the upper 95% confidence limits ranged from 12 (OL) to 36 (OH). These values were used as group-specific cut-off values for analysis.

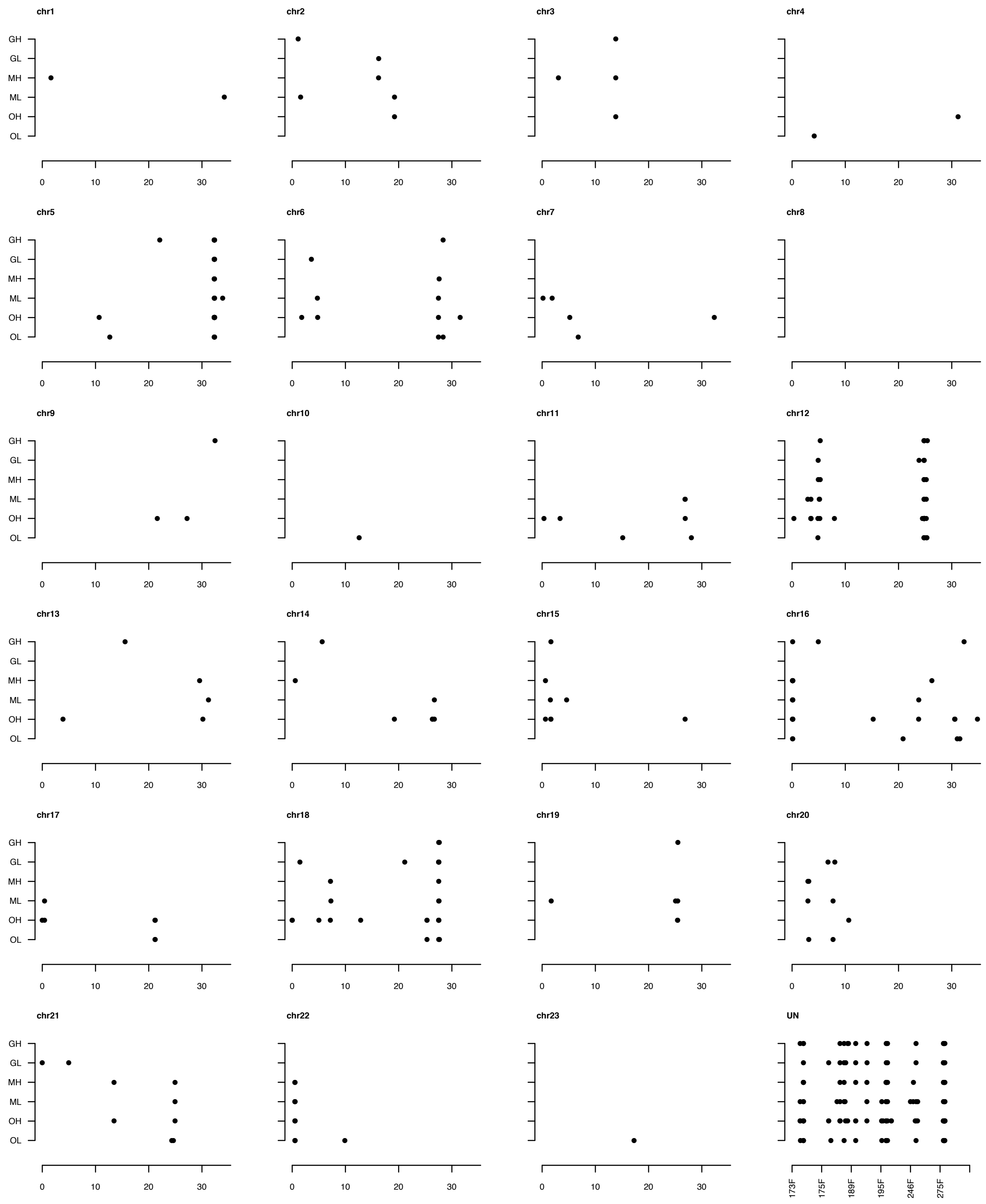

Figure S17: Mapping positions of contigs assembled from male specific k-mers, or y-mers, each population is shown on the y-axis across. Panels for each chromosome, and for six unplaced scaffolds that consistently recruited conitgs.

Table S9: Summary of y-mers and male specific contigs distributions per population sampled. Shown are number and size of contigs and the linkage groups or unplaced scaffolds they map to.

	**GH**	**GL**	**MH**	**ML**	**OH**	**OL**
N Y-mer reads	3546	3892	3378	2710	5828	2277
N Contigs	109	76	92	118	304	95
Total Length of contigs	37863	38489	57583	77196	111551	64488
N50 of contigs	874	913	1104	1041	816	940
N contigs mapped to genome	81	64	80	109	267	85
Length mapped to genome	34342	36173	55528	74644	106566	62521
Length on chr12	3309	3390	5065	6584	11376	7430
%	9.64	9.37	9.12	8.82	10.68	11.88
Length on chr5	3845	4098	2257	8237	9760	5754
%	11.20	11.33	4.06	11.04	9.16	9.20
Length of chr16	1661	0	3176	2060	2543	1829
%	4.84	0.00	5.72	2.76	2.39	2.93
Length on chr18	680	2444	1372	2664	4415	2248
%	1.98	6.76	2.47	3.57	4.14	3.60
Length of chr22	0	0	1447	1853	1718	4570
%	0.00	0.00	2.61	2.48	1.61	7.31
000173F_0	765	689	2185	2603	4304	3929
%	2.23	1.90	3.93	3.49	4.04	6.28
000175F_0	1610	1932	1495	3443	3817	1840
%	4.69	5.34	2.69	4.61	3.58	2.94
000189F_0	676	988	1806	1112	1853	824
%	1.97	2.73	3.25	1.49	1.74	1.32
000195F_0	2972	2919	6002	3354	3526	5363
%	8.65	8.07	10.81	4.49	3.31	8.58
000246F_0	416	702	247	3525	1348	926
%	1.21	1.94	0.44	4.72	1.26	1.48
000275F_0	1637	3088	5450	3881	8135	4199
%	4.77	8.54	9.81	5.20	7.63	6.72

Table S10: Gene annotations for candidate windows on unplaced scaffolds or autosomes (xlsx file)

Investigation of candidate sex-determining regions
Putative protein-coding sequences within contig IV were identified through BlastN searches of the guppy reference transcriptome of Sharma et al (Sharma et al. 2014), using an E-value cutoff of 1e-50. The resulting 273 total matches were reduced to 71 non-redundant matches through an all-against-all BlastN search, retaining only the longest transcript within each cluster of hits. Annotated homologs were identified through BlastX searches of key species on Ensembl 96 (*X. maculatus, D. rerio, X. tropicalis, M. musculus, H. sapiens, D. melanogaster*), using an E-value cutoff of 1e-5. BlastX matches were more frequently observed when searching fish genomes (*X. maculatus* 27/71; *D. rerio* 20/71) than when searching tetrapod or invertebrate genomes (*X. tropicalis* 14/71, *M. musculus* 11/71, *H. sapiens* 12/71, *D. melanogaster* 8/71). Combining results across species, matches were found for 30/71 transcripts; we mostly relied upon annotations from *X. maculatus* (the most closely-related species in the set). Most of the annotated transcripts (19/30) matched known repetitive elements (transposons, retrotransposons, helitrons, retroviruses), while the remainder matched putative "non-repetitive" protein-coding genes. The unmatched transcripts, which tended to be shorter than matched transcripts (IQR of 183–297 nt versus 424–1532 nt), may be non-coding, from highly divergent or de novo protein coding genes, or even artefacts; these were not considered further.

To obtain approximate gene boundaries, the annotated transcripts were mapped onto contig IV using NUCmer (with flags = "-l 10 --maxmatch"). In several cases, multiple transcripts matched common target sequences and mapped to adjacent positions along contig IV, indicating likely split transcripts; boundaries for split transcripts were merged, as needed.

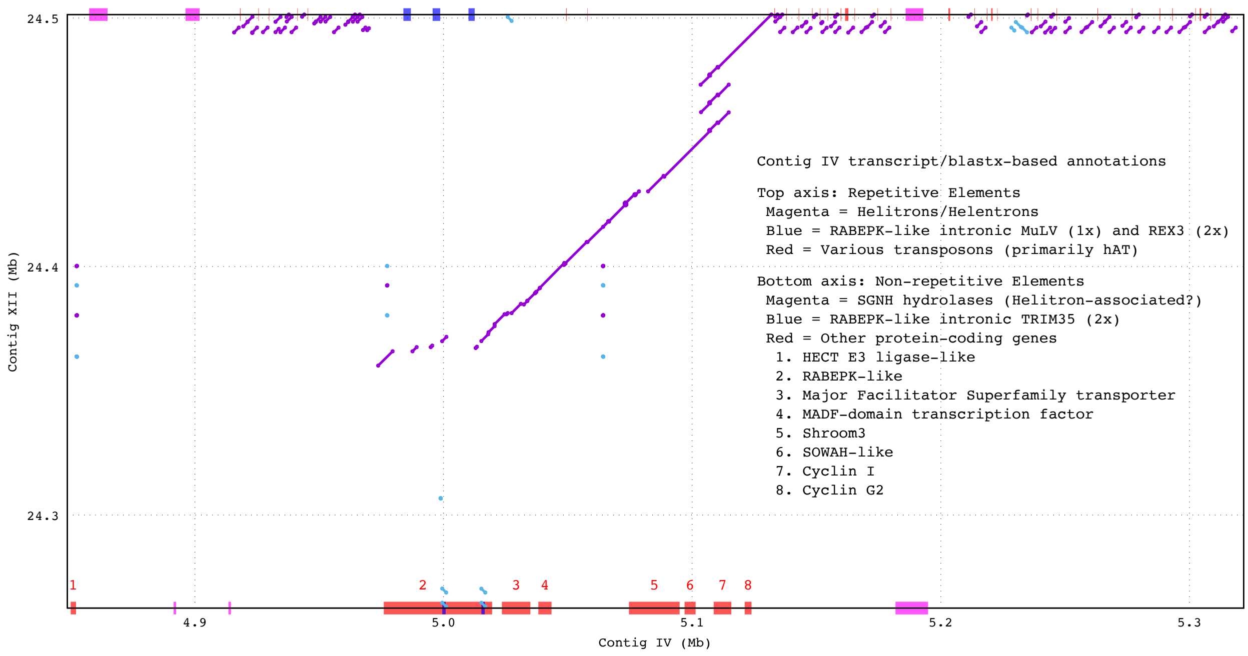

Figure S18: Alignment of contig IV (placed at 5 Mb on LG12) and contig XII (placed at 24 Mb on LG12). Alignment dot plots shown in purple (forward alignment) and blue (reverse alignment). Approximate gene boundaries are shown above and below the figure, with annotation names in insert. The gene-rich region (G1 in the main text) is mostly syntenic between the two contigs, with two notable disrupted sections. One, around 5.0 Mb of contig IV, involves a long TRIM35-containing RABEPK intron (see Figure S19). The other, around 5.1 Mb of contig IV, involves a Cyclin I triplication on contig XII.

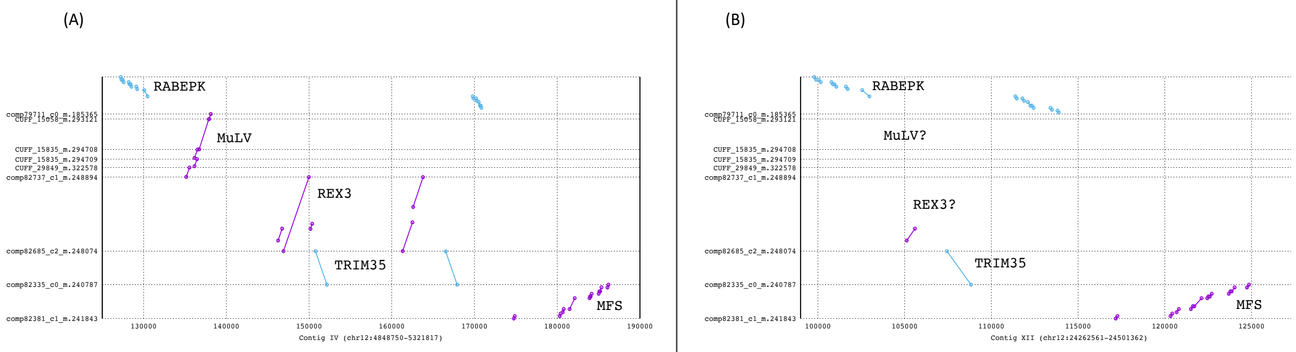

Figure S19: Dot-plot alignments of contig IV (a) and contig XII (b) to RABEPK, MuLV, REX3, TRIM35, and MFS transcripts obtained from the reference transcriptome of Sharma et al. (Sharma et al. 2014). Both contigs contain a RABEPK gene followed by a MFS gene. RABEPK possesses a long intron that, on contig IV (a), contains one MuLV retroelement, two REX3 retroelements, and two TRIM35-like genes. The homologous intron on contig XII (b) contains a single TRIM35-like gene.

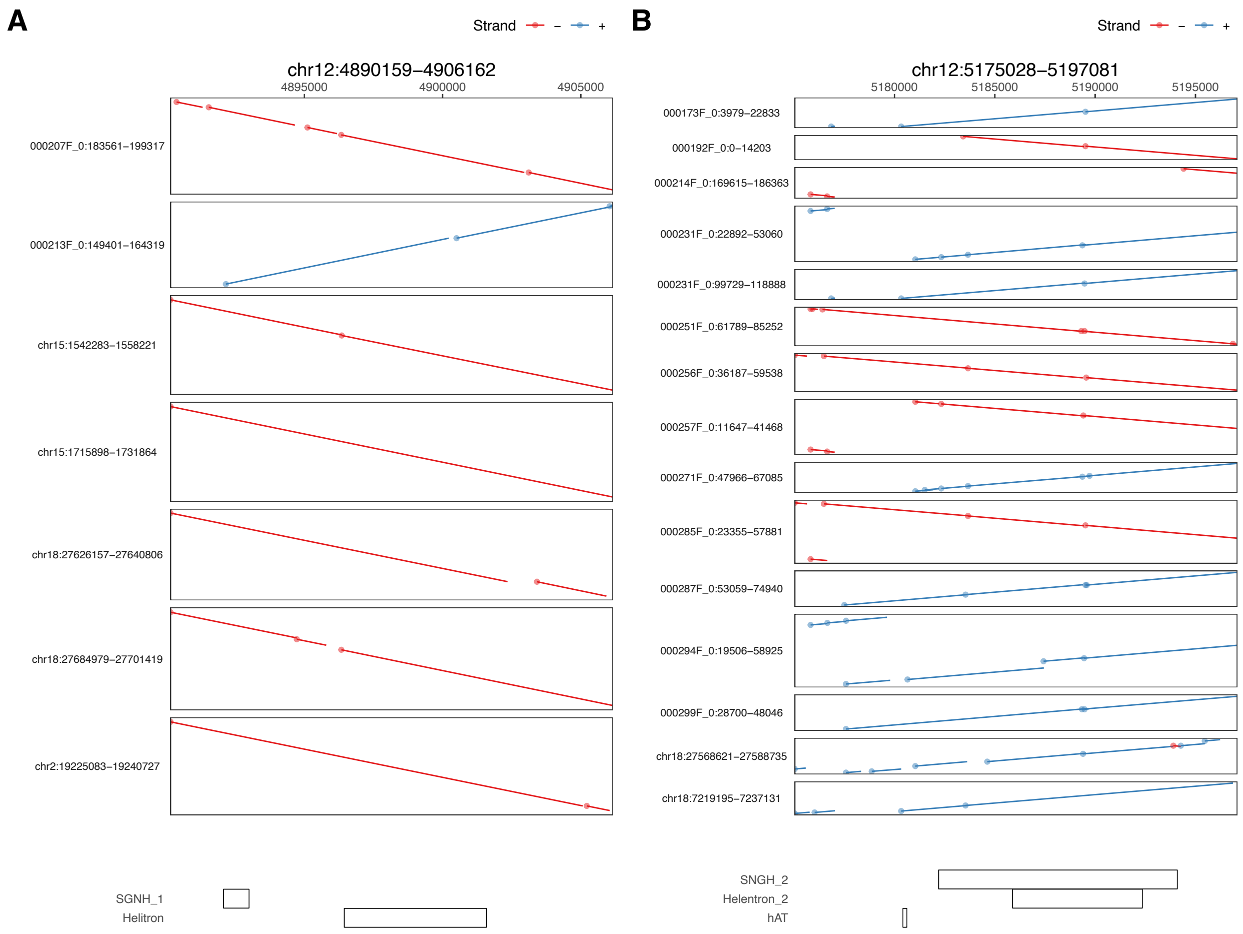

Figure S20: Dot-plot alignments of Texim (SGNH helitron genes) regions from LG12 to other chromosomes and unplaced scaffolds. Below the figure is the position of the Texim genes.

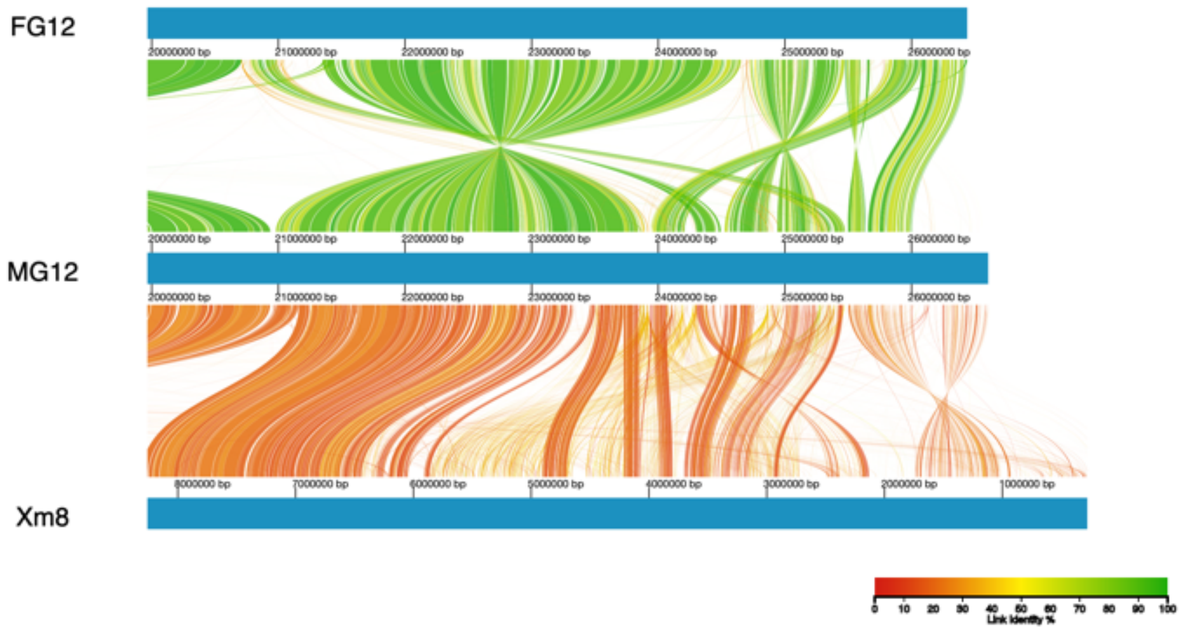

Figure S21: Alignment of the distal end of LG12 between the two guppy genome assemblies (female genome assembly: FG and male genome assembly: MG) and chromosome 8 of *Xiphophorus maculatus*. Colour denotes percent similarity of links (see legend).

Table S11: PCR test for presence of TRIM-35 insertion summarised per sex per population.

Populations	Male Present (Total)	Female Present (Total)
GH	5 (10)	5 (10)
GL	7 (10)	0 (10)
OH	7 (12)	4 (10)
OL	7 (8)	1 (12)
MH	9 (10)	13 (13)
ML	7 (7)	11 (11)

Table S12: qPCR test for TeximY copy number variation, summarised per sex per population.

Populations	Male Present mean Log2(ΔΔCt)	Male mean copy number	Female mean Log2(ΔΔCt)	Female mean copy number
GH	2.44	2.44	0.62	1.02
GL	2.31	2.3	2.02	2.03
OH	2.18	2.19	1.74	1.53
OL	1.89	1.89	1.34	1.23
MH	1.66	1.73	1.14	1.18
ML	1.76	1.77	0.51	1.00

Table S13: PCR-free dataset used for recalibration of SNPs, shown are read and mapping statistics.

Sample	# Raw Reads	# Mapped paired reads	% paired reads mapped	Coverage
GH male11	114,020,460	111,248,558	97.72	34.51X
GH female8	103,069,026	100,424,044	97.62	31.15X
GL male12	121,756,540	118,390,104	97.89	36.33X
GL female5	66,396,712	64,536,388	97.87	19.83X
MH female12	141,133,250	134,769,650	95.72	42.23X
MH male7	134,754,056	128,767,236	96.25	39.82X
OH female19	131,591,146	126,510,152	96.78	39.04X
OH male7	99,474,858	96,144,274	96.79	30.07X
ML female18	155,101,150	149,454,766	97.01	45.90X
ML male16	133,655,092	128,376,742	96.73	39.65X
OL female18	54,255,004	52,271,994	96.49	16.28X
OL male5	62,685,636	60,545,666	96.69	18.89X

Table S14: Primer sequences for PCR and qPCR tests of Table S11 and Table S12, respectively.

Primer Name	Sequence
Trim35 insertion F	CTAAAGATTGCCCACTTCCTG
Trim35 insertion R	TCCTCTGTCCCAGTCCAGAT
TeximY CNV F	AGCAAAACATCCCGGTTATGT
TeximY CNV R	GAGCGTGAAATTGGCCTTCT
Rpl7 F	TCAGAGGTATCAATGGTGTCCC
Rpl7 R	CAGCTTGACAAACACACCGT
